# Regulation of Membrane Association and Downstream Effector Interaction of K-Ras by Its Hypervariable Region

**DOI:** 10.3390/membranes16090304

**Published:** 2026-09-16

**Authors:** Anda Trifan, Hossein Omidi Ardali, Josh V. Vermaas, Till Rudack, Emad Tajkhorshid

**Affiliations:** Theoretical and Computational Biophysics Group, NIH Resource for Macromolecular Modeling and Visualization, Beckman Institute for Advanced Science and Technology, Department of Biochemistry, Center for Biophysics and Quantitative Biology, University of Illinois Urbana-Champaign, Urbana, IL 61801, USA

**Keywords:** peripheral proteins, lipid–protein interactions, Ras proteins, cancer, membrane binding, molecular dynamics simulation, negative lipids

## Abstract

The small GTPase Ras, a central switch in signal transduction of cell growth, is a crucial element in the development of many forms of cancer. Recent studies have shown that membrane association of Ras is essential to its downstream effector recruitment and signal transduction. Here we investigate the membrane association and interaction of K-Ras4B, the most frequently found Ras isoform in tumor cells, using all-atom molecular dynamics simulations totaling 8.5 μs. We find that the isoform-specific, farnesylated hypervariable region (HVR) of Ras plays an important role in the organization and oligomerization of its globular domain (G-domain) on the surface of the membrane. We show that the overall pose of the Ras G-domain on the membrane can be determined by its HVR. The HVR thus can control downstream effector binding by positioning the G-domain on the membrane in a specific orientation. Furthermore, we observe that the HVR alone already transiently dimerizes in the membrane and recruits negatively charged phosphatidylserine lipids around the K-Ras isoform-specific poly-lysine region. The observed HVR dimerization potentially can initiate the dimerization of the full K-Ras, which is essential for downstream effector signaling via various pathways. These atomistic details behind the molecular mechanism of the HVR provide novel insight into Ras interaction with membrane and its availability for downstream effectors.

## 1. Introduction

The Ras (Rat sarcoma) family of proteins are vital regulators of major signaling pathways and key responses to external stimuli in the cell [1]. They are small GTPases mediating the activation of downstream components of cell growth and development when bound to GTP, and inactive otherwise. Constitutively active oncogenic mutations in Ras proteins are found in 30% of all human cancers [2]. Among the three Ras isoforms, H-Ras, N-Ras, and K-Ras (further divided into K-Ras4A and K-Ras4B sub-isoforms), K-Ras4B (from here on, referred to as simply K-Ras) is the most abundant isoform and responsible for 85% of oncogenic mutations [3,4].

Ras activation is regulated by a GTP/GDP molecular switch. GTP binding is the key activation signal for Ras proteins. Deactivation is mediated by the conversion of the bound GTP to GDP, which is facilitated by binding of a GTPase-activating protein (GAP) [5]. Ras protein then recruits a guanine exchange factor (GEF), which significantly lowers Ras affinity for GDP, resulting in GDP unbinding and replacement by GTP.

Ras proteins are composed of two distinguishable parts: the globular, soluble G-domain, which hydrolyzes GTP and is highly conserved across isoforms, and a C-terminal unstructured hypervariable region (HVR), which is usually anchored in the membrane by one or more posttranslational acylations. The G-domain residues are further divided into two lobes, Lobe 1 and Lobe 2 [6]. Lobe 1, *aka* the effector lobe, is largely conserved across all Ras isoforms [7,8] and contains the functionally crucial regions such as the P-loop, switch I, and switch II. Lobe 2, the allosteric lobe, is less conserved and differs slightly across isoforms. The positioning and accessibility of these lobes dictate the ability of a Ras protein to bind to downstream effectors involved in its signaling, or to Ras activators which aid the switch between its active and inactive forms.

A key aspect of Ras biology and pathophysiology is the central role of the cellular membrane in its function. Ras proteins must associate with the membrane to activate their signaling pathways, as the membrane mediates the tight coordination of Ras proteins and their effectors. For example, the membrane has been indicated to mediate the recruitment of GAP and GEFs by Ras proteins [9,10]. The membrane also aids Ras in binding to downstream signaling effectors such as Raf [10,11,12]. As such, studies in the absence of the membrane might not provide an accurate account of the underlying molecular mechanism of Ras proteins.

Lipid-mediated regulation is a hallmark of Ras isoform-specific behavior. Lipid–protein interactions play an important role in signaling and membrane association of Ras proteins, their localization to the plasma membrane [13,14], and in events such as lipid sorting by the HVR [15,16]. Recent studies using complex biomimetic membranes have further demonstrated that membrane lipid composition influences KRAS4B diffusion and nanoscale organization [17]. Furthermore, post-translational modifications such as prenylation, methylation, and farnesylation of the HVR have been shown to anchor the G-domain into the cytosolic side of the plasma membrane in a lipid-dependent manner [18].

The HVR differs significantly across Ras isoforms, maintaining only 10–15% similarity in sequence. In contrast, the G-domains are >90% conserved [19], highlighting that the HVR may be responsible for functional differences between Ras isoforms. Recent analyses of RAS membrane targeting have further emphasized the importance of isoform-specific HVR sequences and lipid modifications in controlling membrane association [20]. K-Ras, specifically, only contains one farnesyl anchor in its HVR, in contrast to N-Ras and H-Ras, which undergo two and three post-translational acylations in this region, respectively. Membrane localization of K-Ras is instead thought to be stabilized by the six consecutive lysines, K175–K180, referred to here as the poly-lysine region, in its HVR [16]. This membrane interaction is believed to be isoform-specific and largely driven by the unique electrostatics of the HVR. As the amino acid sequence and structure of the HVR are tightly correlated to K-Ras function, atomistic details of their interactions with the membrane can bring invaluable insight into K-Ras physiology.

The highly dynamic HVR, especially in the absence of a membrane, is a challenging target for experimental structural determination. Currently, our knowledge of the HVR structure is limited to a few studies reporting full-length crystal structures for Ras proteins, all in the absence of membrane. For example, two structures of K-Ras4B in complex with PDEδ (PDBs 5TAR and 5TB5) show inconsistent conformations: one adopts a short helical HVR segment, while the other displays a flexible loop [21]. A more recent set of full-length K-Ras structures (PDBs 6GOD, 6GOE, and 6GOF) resolve only parts of the HVR, with no consistent secondary structure or membrane interaction [22]. These inconsistencies, due in part to the lack of the membrane context, make it difficult to capture the HVR’s physiological state. These discrepancies highlight the need for modeling the system in the presence of a membrane context to accurately capture HVR’s behavior.

The orientation of the G-domain, and in some cases its dimerization, on the surface of the membrane dictate its ability to recruit and interact with downstream effectors. As such, this aspect of Ras biology has been a target of many studies. Molecular dynamics (MD) simulations [23,24] and smFRET experiments [25] suggest the oncogenic K-Ras G-domain can adopt two main poses on the membrane: one with its three C-terminal α-helices facing the membrane, and the other with its N-terminal β-sheets. Free energy analysis of the kinetic states [26] of membrane-bound K-Ras also details two major orientations in PS:PC membranes. More recently, quantitative paramagnetic NMR combined with computational analysis identified multiple membrane-bound orientations of KRAS4B and showed that HVR-truncated KRAS4B samples a broader orientational range, supporting a role for the HVR in constraining G-domain orientation [27]. Recent work has also demonstrated that the RAS G-domain contributes to the recognition of membrane lipid headgroups and acyl chains, further highlighting the coupling between G-domain orientation and the membrane lipids [28].

A recent study employing coarse-grained (CG) MD combined with various experimental techniques such as NMR proposed a third, “membrane-distal” state in which the G-domain does not even make direct contact with the membrane but is available for Raf binding [29]. Recent studies combining experiments and simulations have shown that membrane lipids modulate K-Ras orientation, diffusion, and nanocluster formation, influencing effector access and dimerization [30,31]. These findings emphasize the importance of membrane context in K-Ras signaling and support the need for atomistic models of how the HVR might govern G-domain behavior.

While these studies reveal much about K-Ras membrane behavior, an atomistic study that focuses on the role of the HVR on the orientation and accessibility of the G-domain in the context of full-length K-Ras is needed. Here, we report all-atom MD simulations systematically studying isolated K-Ras G-domain, isolated HVR, and the full-length protein. Our study shows that the G-domain adopts many different orientations on the surface of the membrane when not tethered to its HVR. The role of the HVR in G-domain anchoring and orientation is further characterized, providing insight into its importance in K-Ras dimerization and function by showing preferential interaction with negatively charged lipids, as well as a secondary anchor by the M170 residue.

The exceedingly high value for K-Ras as an anti-cancer therapeutic target has solicited significant research interest in it, although Ras proteins have been studied for over three decades [32]. Recent advances in structural, biophysical, and computational studies have further highlighted the importance of Ras conformational dynamics and allosteric regulation in controlling interactions with effector and regulatory proteins, with direct implications for therapeutic targeting [33]. Studies combining experiments and multiscale simulations have also shown that membrane lipid composition modulates K-Ras orientation, dynamics, and nanocluster formation, as well as the membrane-associated behavior of K-Ras–RAF complexes [30,34]. Complementary experimental and computational work has further highlighted the coupling between the K-Ras HVR, G-domain dynamics, membrane orientation, lipid composition, and membrane-dependent self-association [35,36,37,38,39]. Currently, there is still a gap in understanding of which Ras structural elements are particularly important to its function. In particular, the role of the HVR in determining membrane interactions and domain orientation has remained elusive. Here, we specifically investigate how the isoform-specific HVR influences the membrane association and orientational behavior of the K-Ras G-domain. By systematically comparing the isolated G-domain, the isolated HVR, and full-length K-Ras, we aim to resolve the contribution of the HVR to K-Ras membrane organization and the resulting accessibility of the G-domain for downstream effector interactions.

## 2. Materials and Methods

### 2.1. Membrane Preparation

Robust signaling activity of Ras proteins depends on their membrane association. Therefore, the interactions of all constitutive G-domain and HVR elements, as well as the full-length K-Ras, with the membrane are of high interest. A summary table with the systems and respective simulation times is provided in Supplementary Table S1. To assess protein–membrane interactions more efficiently, for all systems we performed simulations using the accelerated HMMM membrane model [40], with the exception of the part concerning the full-length K-Ras for which a full membrane was used (see below). HMMM is a membrane model with short-tailed phospholipid tails [41] and faster lipid diffusion that allows for enhanced sampling of lipid–protein interactions at reduced timescales, while retaining atomistic presentations on the surface of the membrane [42,43,44,45,46,47,48].

For all simulations in this study, the HMMM membranes were built using the HMMM BUILDER [49] tool in CHARMM-GUI, ref. [50] with a composition of 70:30 phosphatidylcholine (PC): phosphatidylserine (PS), a commonly used composition for both experimental and computational K-Ras studies [29,51,52,53]. The membranes contained 140 lipids in each leaflet (42 PS, 98 PC), resulting in membranes of 110 × 110 ${Å}^{2}$, with the membrane core filled with 1584 solvent (dichloroethane; DCLE) molecules. To maintain membrane shape, harmonic restraints (k=0.05 kcal mol^−1^${Å}^{-2}$) were applied along the *z* direction (membrane normal) to the acyl tail carbonyl carbons (C21 and C31) of all phospholipids. To prevent leaking of DCLE molecules into the aqueous phase, half-harmonic potentials were applied when |z|>20 using the GridForce [54] feature of NAMD [55,56].

### 2.2. Membrane Binding of G-Domain Alone

In order to determine stable interactions between the G-domain and the membrane, six independent systems of G-domains in the presence of HMMM membranes were simulated. Each system included two G-domains (PDB 4OBE), whose bound GDP nucleotide was aligned and replaced with GTP before the simulations. The GTP replacement generated a nucleotide-bound state consistent with active KRAS, but no constraints were imposed on the switch regions. The proteins were capped with an N-terminal ammonium and a C-terminal carboxylate group. The proteins in each system were rotated about the *x* and/or *y* axis by 15–30 degrees to deliberately randomize their initial orientations and ensure less biased sampling of membrane-binding configurations, and were placed ∼125 Å from each other (COM-COM distance) and ∼25 Å from the cis leaflet of the membrane. The PSFGEN plugin of VMD (Visual Molecular Dynamics) [57] was used to assemble the protein–lipid systems. They were solvated using the SOLVATE plugin of VMD and neutralized with Na^+^ and Cl^−^ ions for a concentration of 150 mM NaCl with AUTOIONIZE. The resulting systems consisted of ∼138,000 atoms and box sizes of ∼115 × 115 × 142 ${Å}^{3}$. The systems were energy-minimized using the steepest descent method for 500 steps, followed by a 3 ns equilibration protocol restraining the protein heavy atoms, Mg^2+^, GTP molecules, the DCLE solvent, and the C21, C31 atoms of the phospholipids with a force constant of k=0.5 kcal mol^−1^${Å}^{-2}$. All restraints were then removed and a production run of 1 μs for each system was performed, totaling 6 μs of membrane-binding simulations of the G-domain alone. No external forces or positional biases were applied to drive membrane association, allowing the G-domain to interact with the membrane through spontaneous diffusion alone.

### 2.3. Membrane Binding of HVR Alone

The HVR sequence and structures are significantly different in different Ras isoforms, likely affecting their function and membrane interaction. The HVR is thought to be unstructured, and so we developed 24 different models for the K-Ras4B HVR (residues 166–185), which includes the linker region later implicated in dimerization, via the Rosetta ab initio structure prediction protocol [58]. Rosetta utilizes a local fragment library of solved structures in the Protein Data Bank as a starting point, and then iteratively exchanges fragments using a Metropolis Monte Carlo protocol to minimize a knowledge-based scoring function Kaufmann et al. [59]. From an initial pool of 992 Rosetta models, we selected 24 representative low-energy structures to maximize structural diversity for subsequent simulations. Each of the HVRs was capped with an N-terminal acetyl capping group and a C-terminal methyl acetate group. The HVRs were also farnesylated at C185, as would be the case in the full-length K-Ras4B protein (KRAS4A contains an additional palmitoyl anchor and was not modeled here).

We set up three membrane simulation systems, each including eight HVR structures positioned above the membrane—four per leaflet—with their farnesyl groups oriented toward the bilayer to guide insertion during the simulation. This arrangement allowed spontaneous HVR–HVR encounters to be monitored and potential self-association/dimerization events to be quantified without bias. The HVRs were initially placed ∼5–8 Å away from each leaflet (top and bottom), ∼60 Å apart laterally, and ∼100 Å diagonally from each other. The parameters for the farnesyl group were obtained by analogy from the CHARMM36 [60] isoprene units, based on prior work Vermaas et al. [61]. The linkage to the cystine residue was similarly taken by analogy through combining the CHARMM36 lipid and protein force fields [60,62]; the choice of CHARMM36 rather than CHARMM36m was made to maintain compatibility with previously validated KRAS HVR parameters, although CHARMM36m arginine updates are not expected to qualitatively alter the membrane anchoring results here.

The combined HVR-membrane systems were solvated and neutralized with 150 mM KCl. The resulting systems contained 150,000–177,000 atoms and box sizes of approximately 115 × 115 × 150 ${Å}^{3}$.

Similar to the G-domain membrane-binding simulations, the HVR systems were energy-minimized using steepest descent for 500 steps, followed by a 3-ns equilibration restraining the protein heavy atoms, the farnesyl tail, the DCLE solvent, and the C21, C31 atoms of the phospholipids (k=0.5 kcal mol^−1^${Å}^{-2}$). These restraints were then removed and a production run of 0.5 μs for each system was performed.

### 2.4. Membrane Binding of Full-Length K-Ras

After studying the constitutive functional domains of K-Ras, i.e., the G-domain and the HVR, separately, the full-length protein (residues 1–185, farnesylated) was constructed and simulated to observe how the interaction of the G-domain with the membrane might change in the presence of the HVR. The HVR configuration used to construct the full-length K-Ras system was selected based on the solvent-accessible surface area (SASA) of the N-terminal K166. Specifically, SASA was used to identify a membrane-bound HVR conformation in which K166 was maximally solvent accessible, thereby facilitating its connection to the C-terminal R165 of the G-domain without introducing steric clashes. The system was solvated and ionized as before. The newly generated full-length protein was minimized and equilibrated as above (500 steps of minimization and 3 ns of equilibration), followed by a production run of 500 ns with HMMM lipids. Subsequently, the last frame of this simulation was converted to a full-tailed membrane using PSFGEN. The resulting system was energy-minimized for 500 steps and equilibrated with NAMD for 3 ns before being transferred to the Anton2 supercomputer and simulated for an additional 500 ns using default Desmond conditions (e.g., with 2.5 fs timesteps). The simulation steps are outlined in Results.

### 2.5. Simulation Protocol

Unless stated otherwise, all simulations were performed using NAMD2 (v2.14) [55,56], TIP3P water [63] and the CHARMM36 force-field parameters for lipids and proteins [60,62] in an NPAT ensemble, and using a 2-fs timestep. Temperature was maintained at 310 K using the Langevin thermostat with a damping coefficient γ = 0.5 ps^−1^, while the pressure was kept constant using the Nosé-Hoover Langevin piston method [64,65]. A cutoff of 12 Å and a switching distance of 10 Å were used to calculate non-bonded forces. The particle mesh Ewald (PME) method [66] was used to calculate long-range electrostatics. ConstantArea in NAMD2 was used for the HMMM simulations to maintain headgroup densities.

### 2.6. Analysis

We defined a protein–lipid contact when any heavy atom of the protein was within 3.5 Å of any heavy atom of the lipids. To compare binding interactions of different regions of the protein, these contacts were mapped separately for the effector lobe, allosteric lobe, and HVR, consistent with key functional distinctions. The resulting contacts are presented for each protein in each simulation system. Contacts to different protein parts are highlighted in this figure for easier identification of the functional parts of K-Ras interacting with the membrane: effector lobe (yellow), allosteric lobe (red), and the HVR (teal).

To characterize the orientation of the G-domain on the membrane, we defined two angles: (1) the “GTP” angle ($\theta_{\mathrm{GTP}}$), calculated between the membrane normal and the vector connecting the G-domain center of mass (COM) to the COM of GTP; and (2) the “Helix” angle ($\theta_{\mathrm{Helix}}$), defined as the angle between the membrane normal and the vector connecting the G-domain COM to the COM of helix α5. These angles provide a simplified yet informative projection of orientation space.

Since we observed multiple membrane-bound orientations, we performed clustering to classify them. The data from all G-domain simulations were concatenated and analyzed using the Gaussian Mixture Model (GMM) algorithm with the scikit-learn package [67]. The appropriate number of clusters to use was determined by calculating the fraction of variance explained (FVE). This calculation was repeated 10 times for up to k=20 clusters. The kink in the curve, which is approximately at k=7, represents the optimal number of clusters for the GMM analysis.

The secondary structure of the HVR throughout the trajectories was evaluated by the Timeline plugin of VMD and plotted using Matplotlib 3.8.3 [68]. To characterize the membrane binding mode for each HVR, the *z*-distances between the COM of individual HVR residues from the *cis* phosphate plane were used. These pairwise distances were then clustered using the *k*-medoids clustering algorithm [69] to characterize distinct poses that HVR assumes on the surface of the membrane. The clusters were projected using principal component analysis (PCA) [67] and plotted using the first two PCs to reduce the dimensionality of the initial dataset and aid visualization. Two resulting clusters were identified from the HVR-only systems based on the z-distance between HVR residues and the membrane phosphate plane, and their respective centers (medoids) were calculated, providing two distinct representative types of HVR-membrane interactions. We further quantified HVR–HVR associations by computing inter-HVR contact frequency across all trajectories and visualizing distance distributions and contact lifetimes. To assess the HVR–lipid interactions, radial distribution function g(r) calculations were performed between the side-chain nitrogen atom of lysines and the phosphorus atom of lipids, using the *measure gofr* function in VMD with a *delta* value of 0.1 Å and an *rmax* of 10 Å. The contacts within 3.5 Å between HVRs (inter-HVR contacts) as well as those between HVRs and lipids were calculated separately and normalized to the maximum number of contacts observed across the simulations.

The contacts within 3.5 Å between HVRs (inter-HVR contacts) as well as those between HVRs and lipids were calculated separately and normalized to the maximum number of contacts observed across the simulations.

## 3. Results and Discussion

To characterize the structural organization of K-Ras on the membrane, we analyzed the conformations resulting from interactions between lipid bilayers and either the isolated G-domain, the isolated highly dynamic hypervariable region (HVR), or the full-length K-Ras protein. We present a comprehensive set of simulations, including 12 distinct G-domain orientations, 24 different HVR configurations, and one full-length K-Ras system embedded in simplified HMMM membranes [41] composed of 70:30 PC:PS lipids, as well as in full-tail membranes for comparison.

To evaluate the role of the HVR in G-domain membrane orientation, we compared the orientation of the G-domain in systems with and without a covalently attached HVR. In the first section, we analyze how the isolated G-domain interacts with the membrane by clustering its orientation across simulations.

Next, we examine the dynamics of the HVR in isolation, focusing on negative lipid recruitment driven by its isoform-specific poly-lysine motif and its transient, spatially restricted associations with neighboring HVRs. We also apply clustering analysis to identify two distinct membrane-bound conformations of the HVR, differentiated by their depth and interaction profiles.

Finally, we analyze full-length K-Ras to determine how HVR tethering modulates G-domain orientation on the membrane. This comparison reveals how different domains of K-Ras work cooperatively to establish membrane binding configurations and support a mechanistic model in which the HVR acts as both an anchor and modulator of G-domain positioning.

### 3.1. Characterizing G-Domain–Membrane Interaction

Signaling activity of K-Ras is dictated by both GTP binding and its obligatory association with the membrane [10,11]. The orientation and interactions of the G-domain on the membrane surface determine the ability of the Ras protein to bind to effectors and, subsequently, conduct downstream signaling.

**Figure 1:**
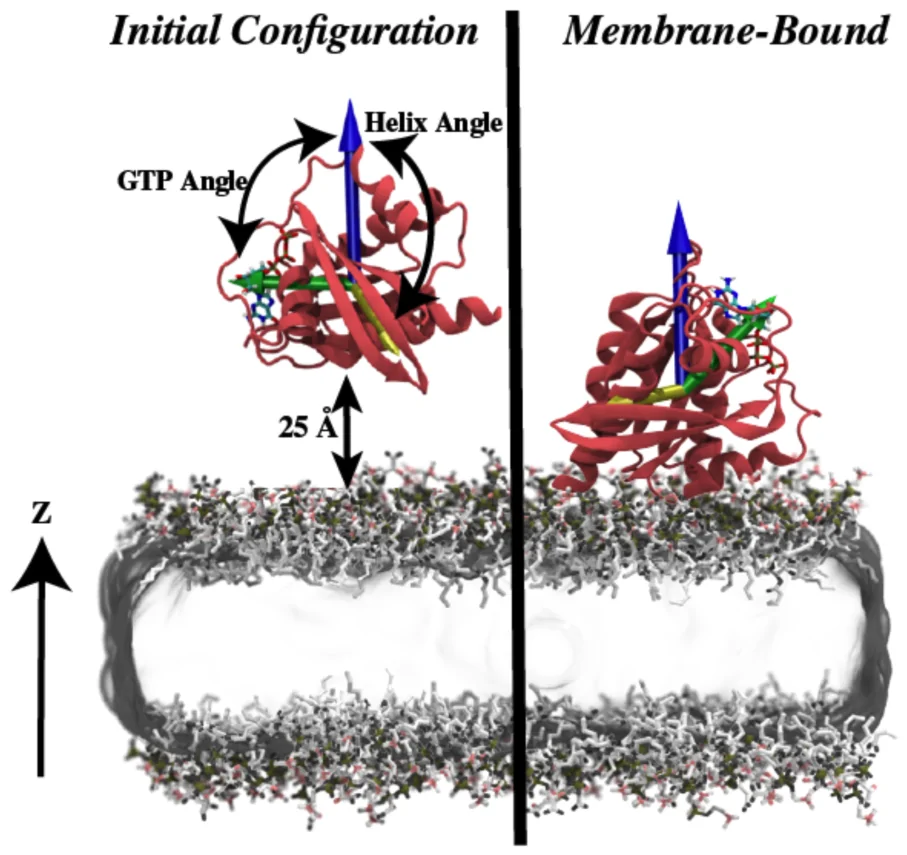
**G-Domain simulation setup.** Example of an initial G-domain configuration with truncated HVR, placed approximately 25 Å above an HMMM membrane (**left**), and a representative membrane-bound orientation obtained from HMMM simulations (**right**). Vectors used to define orientation angles are colored as follows: membrane normal (blue), vector to GTP (green), and vector to helix α5 (yellow).

To investigate the atomistic interaction of the G-domain at the membrane interface, we performed six simulations (replicas R*x*) of systems containing two truncated K-Ras G-domains (P*x*; residues 1–165) lacking the HVR (Figure 1).

**Figure 2:**
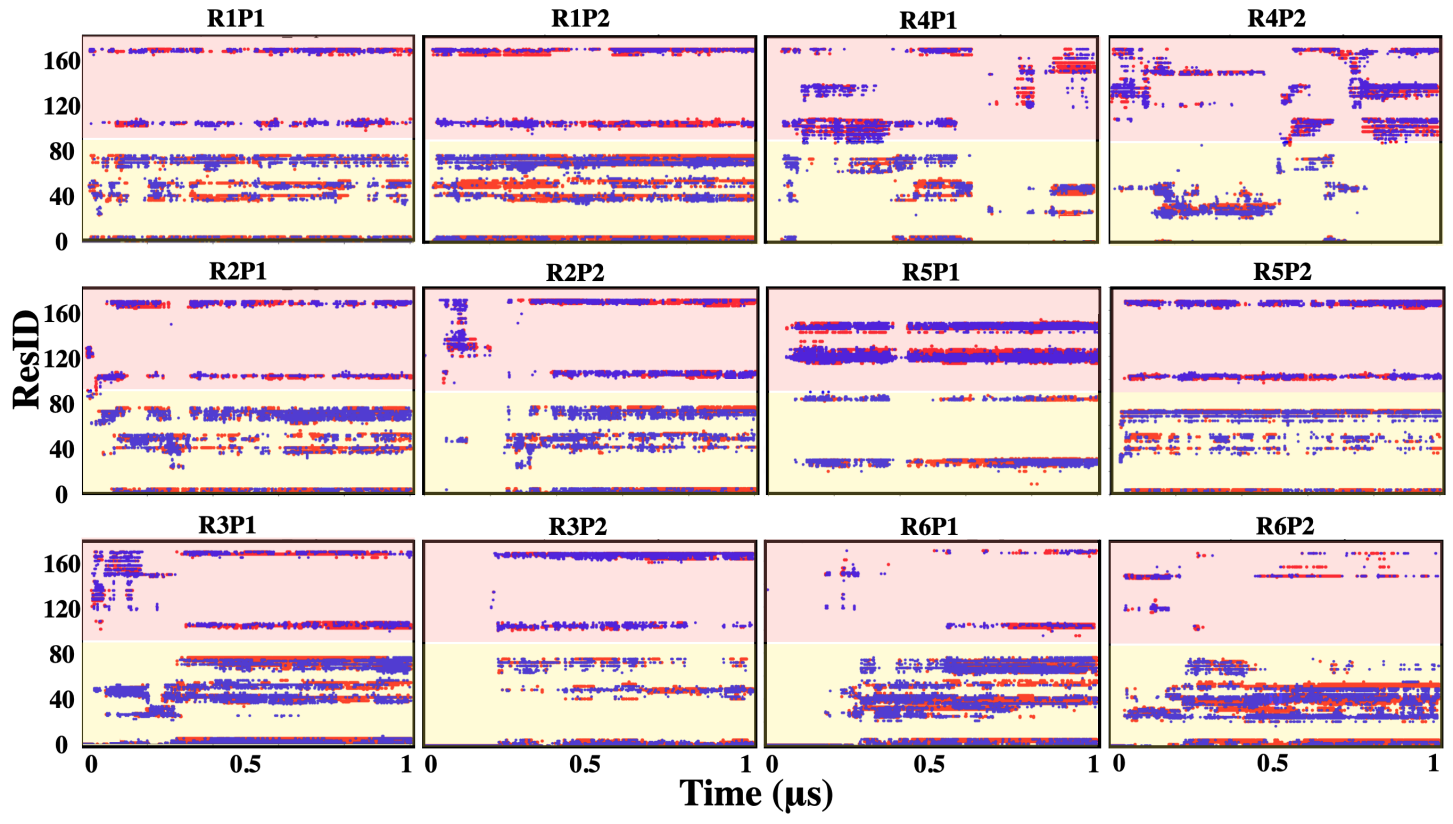
**Membrane contacts of the K-Ras G-domain without HVR.** Time progression of the K-Ras–lipid interaction for the truncated G-domain. Protein interactions with PC lipids are indicated in blue, and with PS lipids in red. A contact is defined as a heavy atom of the protein within 3.5 Å of any heavy atom of a lipid. Highlighted are the effector lobe (yellow) and the allosteric lobe (pink). Membrane interactions are distributed across both lobes of the G-domain, reflecting heterogeneous and dynamic binding.

In order to characterize the binding of the G-domain to the surface of the membrane we monitored the contacts between the proteins and lipids (Figure 2). All G-domains established contact with the membrane within the first 100 ns, although without a consistent set of residues. In 83% of the replicas, the G-domains remained membrane-bound throughout the 1 μs simulations. In the remaining cases (R4P1, R4P2), they transiently detached before rebinding via a different interface (Figure 2). Despite the membrane being PC-rich, we observed significant contacts with PS lipids, suggesting a possible electrostatic preference for negatively charged lipids.

**Figure 3:**
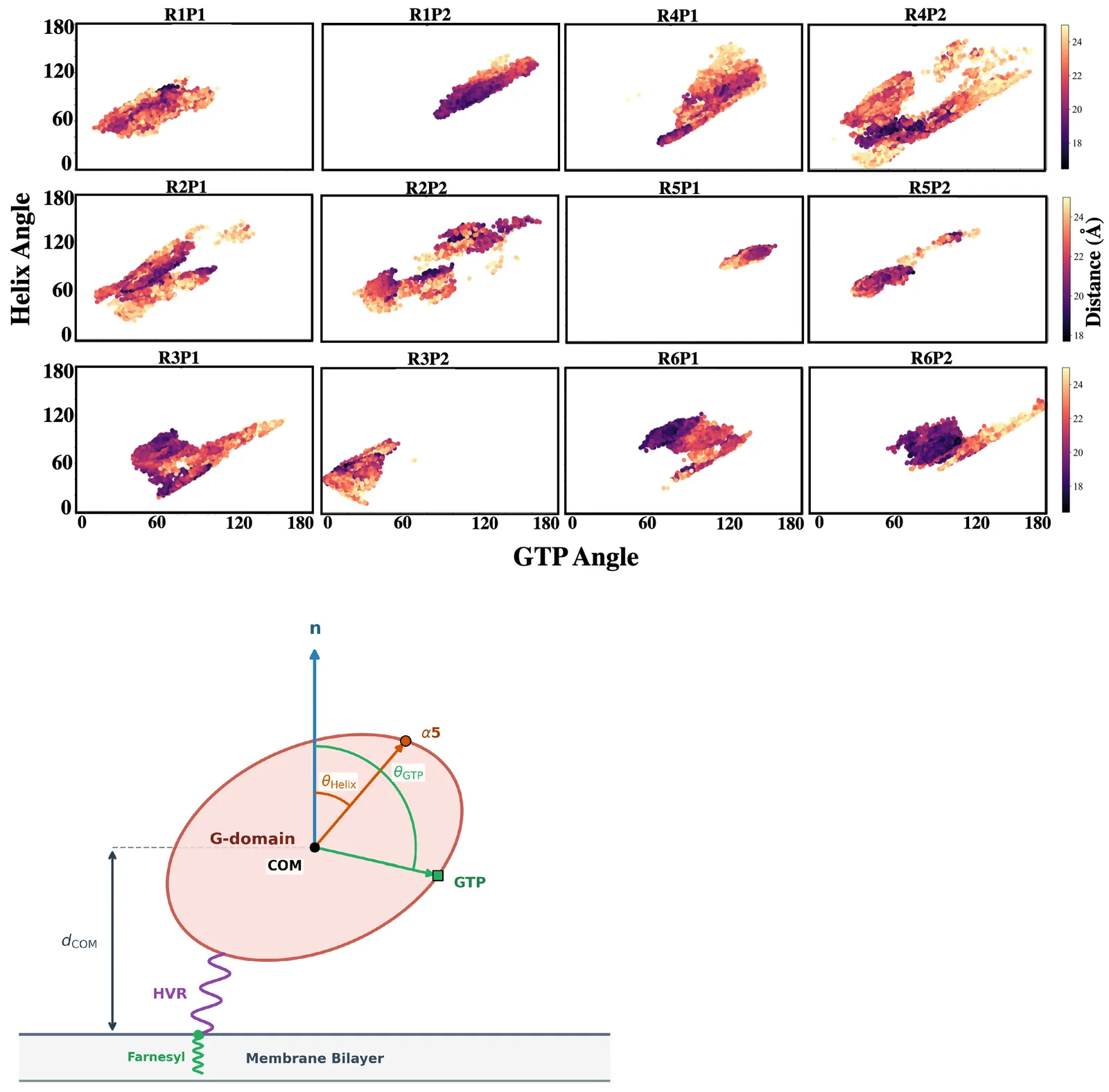
**Orientation of the K-Ras G-domain without the HVR on the surface of the membrane.** Each point represents the G-domain’s orientation over time in the space defined by the GTP and Helix angles and is colored by its COM distance from the membrane phosphate plane. Darker colors indicate closer membrane association. The schematic below illustrates the definition of the GTP and Helix angles relative to the membrane normal (*n*), as well as the distance ($d_{\mathrm{COM}}$) between the G-domain center of mass (COM) and the membrane phosphate plane.

To characterize G-domain orientation, we defined two angles between G-domain vectors and the membrane normal (Figure 1), and tracked them throughout the simulations. The G-domains initially diffuse above the membrane, then adopt stable orientations (Figure S1). The availability of the effector lobe and its functional constituents determine downstream binding, therefore the orientations were analyzed for the orientation of the membrane-bound G-domain to see if the conformations would allow for such interactions (Figure 3).

Among the 12 G-domains, we observed diverse membrane-bound poses with no apparent correlation to the starting configurations, suggesting no strongly preferred orientation.

While most G-domains sampled one dominant orientation, some (e.g., R4P2) transitioned between multiple distinct poses.

#### Clustering of Membrane-Bound States

**Figure 4:**
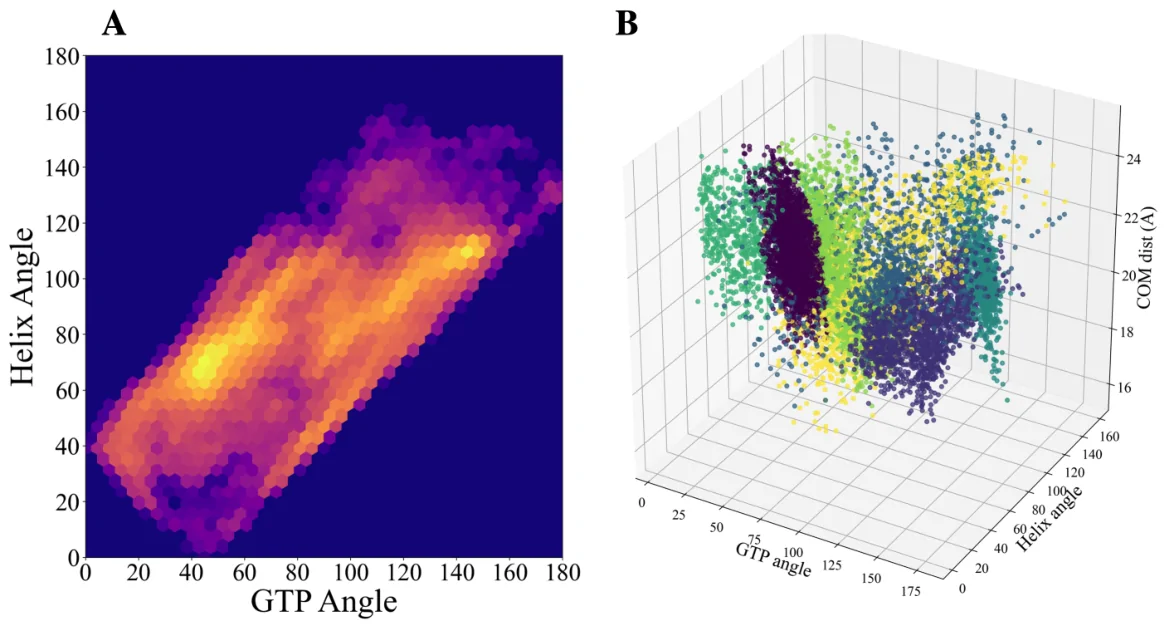
**Clustering the orientation of membrane-bound K-Ras G-domain without HVR**. (**A**) Orientation data from all 12 simulation replicas (see Figure 2) was merged. Bin colors range from low (purple) to high (yellow) frequency of occurrence. (**B**) Gaussian Mixture Model (GMM) clustering reveals seven distinct membrane-bound orientations sampled by truncated K-Ras.

To quantitatively assess the orientations adopted by the G-domain on the membrane, we employed Gaussian Mixture Model (GMM) clustering on the combined data from 12 simulation replicas. These simulations sampled orientations using two angles defined in Figure 1, enabling a systematic comparison of membrane-bound poses across all replicas. Clustering revealed seven distinct orientation clusters (Figure 4B), confirming that the truncated G-domain engages the membrane through multiple preferred binding modes. These results indicate that membrane association by the G-domain alone is inherently heterogeneous, with no dominant or persistent configuration.

Consistent with this variability, we observed that individual replicas displayed divergent behaviors. Some maintained stable membrane contact via the effector lobe, while others detached and reoriented to rebind through different residues. This highlights a lack of memory of initial membrane contact and suggests that electrostatic interactions alone are insufficient to anchor the G-domain in a consistent orientation. This dynamic rearrangement is functionally significant. Without the HVR, the G-domain exhibits low specificity and high rotational freedom on the membrane surface, which likely reduces the probability of maintaining a productive interface for effector binding. This finding supports the idea that additional elements, such as the HVR or partner proteins, are needed to stabilize G-domain orientations conducive to signaling. Future work could improve this clustering analysis by incorporating comparisons with randomized baselines or null models, allowing a more rigorous assessment of orientation enrichment and sampling preferences. Taken together, these results show that, in the absence of the HVR, the G-domain samples heterogeneous membrane-bound orientations rather than a single persistent configuration, providing a reference for assessing the orientational restriction observed in full-length K-Ras.

### 3.2. Membrane Binding of HVR as a Critical Step in Ras Signaling

Given the unpredictable behavior of the G-domain on the surface of the membrane, the HVR appears to offer an account for the significant difference in Ras isoforms, dictating where Ras localizes and functions. Studying the HVR experimentally is challenging due to its highly disordered nature. Nevertheless, atomistic details about HVR are needed due to their potentially critical role in embedding K-Ras into the membrane, and consequently enabling signaling partners through downstream effector binding.

**Figure 5:**
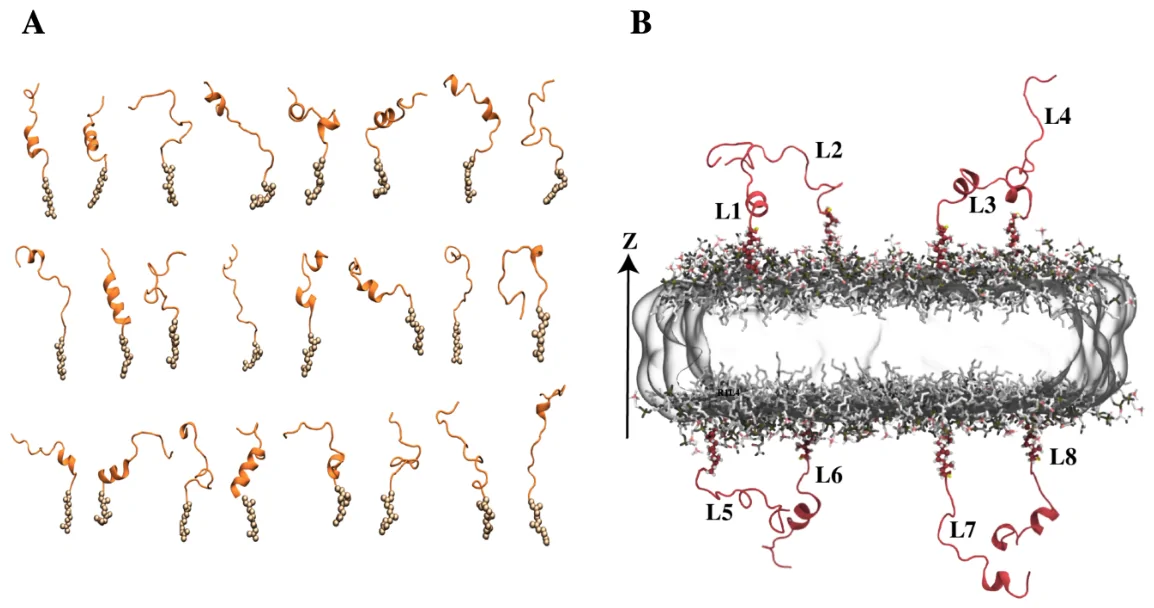
**Initial HVR structures and the HVR simulation setup**. (**A**) These HVR structures were positioned close to the membrane and used to initiate three simulations of membrane-bound HVRs. (**B**) Example shown is the initial system R1. Eight HVR structures were placed initially in each simulation system (four above the membrane and four below it).

Here, we simulated 24 ab initio conformations of the HVR with varying initial secondary structures in the presence of membranes (Table S1). Some HVR structures are completely disordered, while others have modest helical content, varying in both location and length (Figure 5A). The selected HVR conformations were distributed among three membrane simulation systems, with eight HVR structures initially placed in each system (Figure 5B). The secondary structure of the HVRs remains dynamically variable, with some forming sporadic helical content over the trajectories, while others maintaining a random loop form (Figures S2 and S3).

#### 3.2.1. Modes of Insertion of the HVR into the Membrane

**Figure 6:**
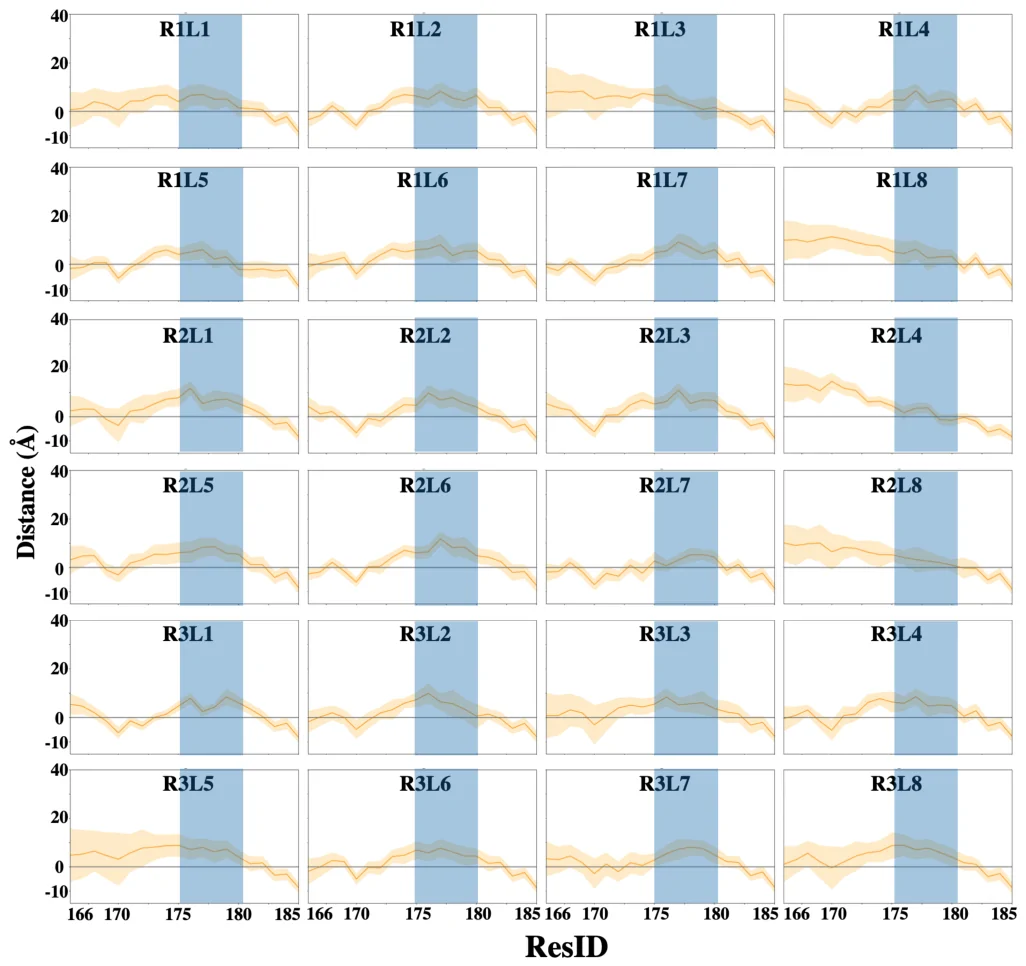
**HVR interaction with the membrane**. The modes of interaction between the HVRs and the membrane lipids are shown. Each RnLm plot shows the distance between the COM of each side chain in the HVR to the phosphate plane of lipids (horizontal line at 0) in simulation replica Rn and HVR Lm. Orange solid line represents the average values, while the orange shaded area represents the standard deviation throughout the trajectory. The blue shaded areas highlight the poly-lysine chain.

With a large ensemble of HVR structures simulated, we could analyze the HVR interaction with the membrane and how its side chains lie on, or insert into the membrane. While the farnesylated C-terminal of all HVRs inserted into the membrane, the N-terminal residues are solvent-exposed and free to diffuse or interact with the membrane. By analyzing the distance between the COM of each side chain to the phosphate plane (Figure 6), we observed patterns of two distinct membrane-bound states. In one state, the N-terminus is above the membrane plane, diffusing in solution, as shown by the large standard deviation in its position throughout the trajectories (e.g., R1L3 in Figure 6). In the other form, which is a more commonly observed state, the N-terminus is less flexible, as the hydrophobic residue M170 inserts into the membrane, acting as a second anchor. The poly-lysine chain remains above the phosphate plane, which is to be expected, as the positively charged lysines form strong electrostatic interactions with the negatively charged PS head groups.

**Figure 7:**
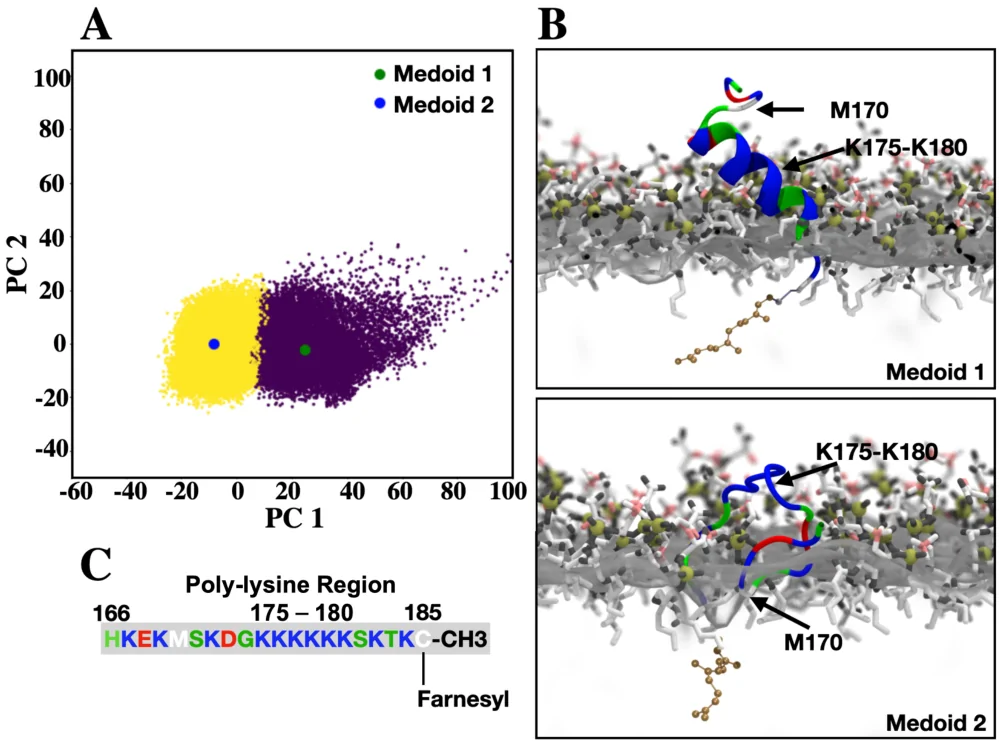
**Clustering of HVR membrane binding modes and representative structures.** (**A**) Principal components (PC) of the dimensionally reduced data represented in Figure 6 clustered into binding. modes colored with yellow and purple and their representative centers (Medoid 1: green, Medoid 2: blue). For each binding mode, a representative structure is shown for the respective medoid (**B**). The HVR assumes two distinct poses on the surface of the membrane: one with N-terminus residues exposed to the solvent (from R1L8 at t= 286 ns), and another one where they are buried into the membrane with the hydrophobic residue M170 (from R1L8 at t= 328 ns). (**C**) The primary sequence of K-Ras HVR, with amino acids colored by type (blue: basic, red: acidic, green: polar, white: hydrophobic).

The *k*-medoid clustering analysis of the pairwise distances between the side-chain COMs and the phosphate plane also results in two well-defined populations (Figure 7A). PCA was also performed for ease of visualization, and the first two PC components are shown for the clustering results (Figure 7A). The centers of the clusters (i.e., the medoids) are subsequently chosen as representative structures for the clusters, showing two distinct membrane-binding poses (Figure 7B). The corresponding atomistic snapshots from the trajectories for the two medoids reveal the distinct position of the M170 hydrophobic residue, either exposed to the solvent or buried into the membrane. Thus, the HVR adopts two principal membrane-bound configurations distinguished by whether M170 remains solvent-exposed or inserts into the membrane as a secondary anchor.

#### 3.2.2. Anionic Lipid Recruitment by HVR

**Figure 8:**
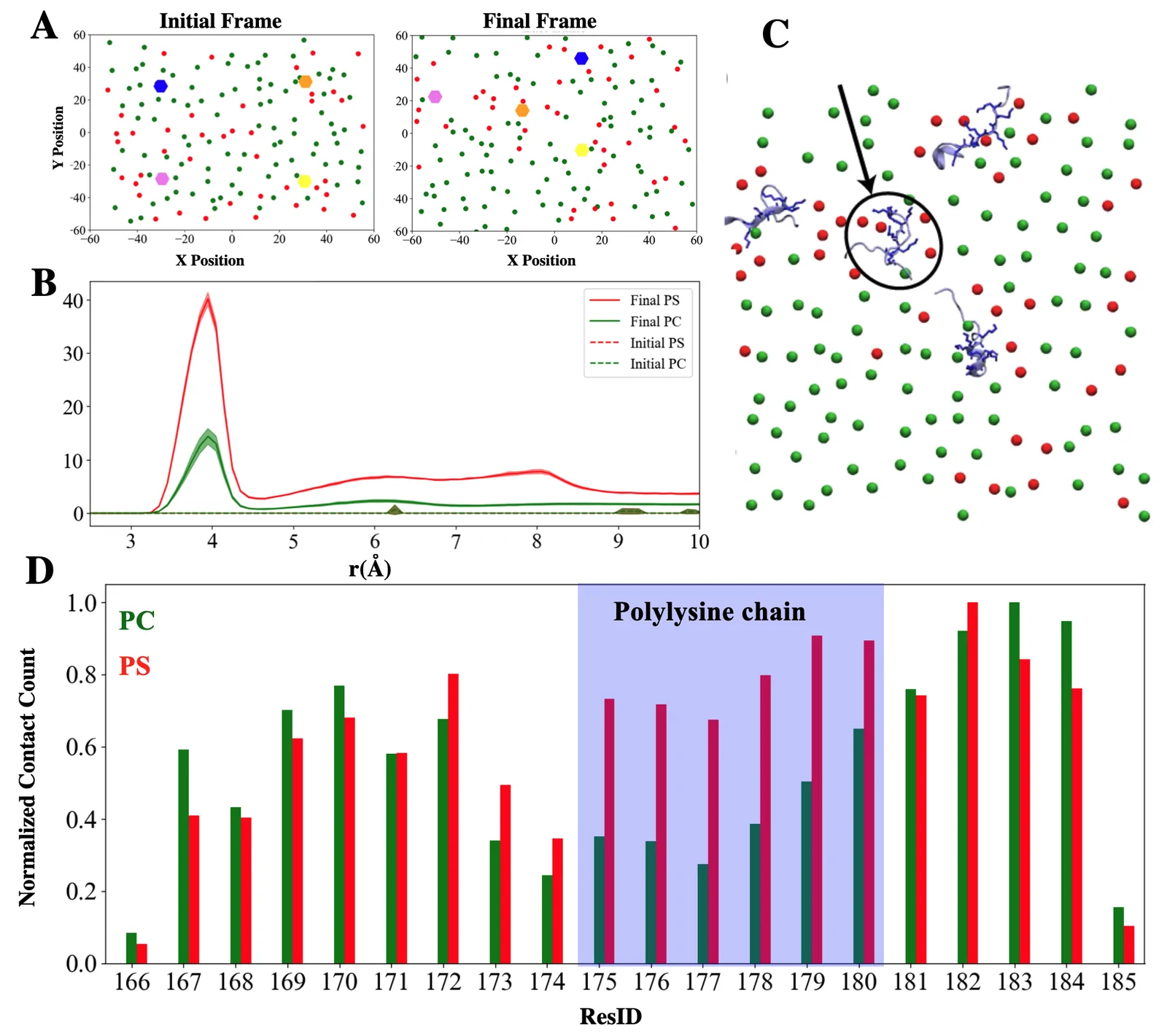
**Lipid interaction of HVR.** (**A**) In initial simulation setup, the different lipids (PS:red dots; PC:green dots) are randomly distributed as shown in the 2D xy map of the membrane. During the simulations, the PS lipids aggregate around the HVRs (hexagons), leading to a well-ordered localization. (**B**) The radial distribution of lipids with respect to HVRs for all three systems supports the aggregation of PS around the HVRs. Comparing the initial g(r) for PS and PC (dashed lines) with final ones (solid lines) clearly shows aggregation of lipids, especially PS around HVRs. (**C**) A representative snapshot showing PS (red) lipid aggregation around the HVRs (purple). (**D**) The positively charged poly-lysine chain (residues 175–180) has substantially more interactions with the negatively charged PS lipids (red bars) than the PC lipids (green bars). A contact is defined as a distance of 3.5 Å or shorter between the HVR residues and the lipid heavy atoms. The values are normalized to the maximum number of contacts. The poly-lysine chain of the HVR leads to a local aggregation of PS lipids around the HVR.

K-Ras HVRs undergo post-translational modifications, during which residue C185 is methylated and farnesylated, thereby increasing the lipophilicity of the HVR. In the majority of our simulation set, the farnesyl tails are inserted into the membrane within the first 50 ns, similar to timescales observed for free lipid insertion into the membrane [42]. The other two isoforms of Ras, N- and H-Ras, have multiple lipidation sites to stabilize their membrane attachment. K-Ras compensates for its single farnesylation site with a positively charged poly-lysine chain (Figure 7C), which can favorably interact with anionic lipids such as PS. Randomly dispersed PS lipids (Figure 8A) diffuse during the simulations, leading to an aggregated PS arrangement around the HVRs (Figure 8C). The lysine side chains in the poly-lysine chain preferentially interact with anionic lipids (Figure 8D). The preferred interaction with PS lipids is also evident from the evolution of the pair distribution function (g(r)), which shows a significantly higher density of PS lipids near the poly-lysine chain.(Figure 8B). The electrostatic interactions arising from a single positively charged lysine are not sufficient to drive the PS clustering around the HVR, as can be observed by the number of interactions between each amino acid and the two types of lipids (Figure 8D) Residues K167, K169, K182 and K184 form contacts with PC and PS without a significant preference toward one or the other. The interaction with PS lipids in the plasma membrane is required for K-Ras to signal, as the depletion of PS lipids dissociates K-Ras from the membrane [70]. Therefore, the poly-lysine chain forms indispensable lipid interactions and is essential for K-Ras membrane association and function.

#### 3.2.3. HVR–HVR Interactions

**Figure 9:**
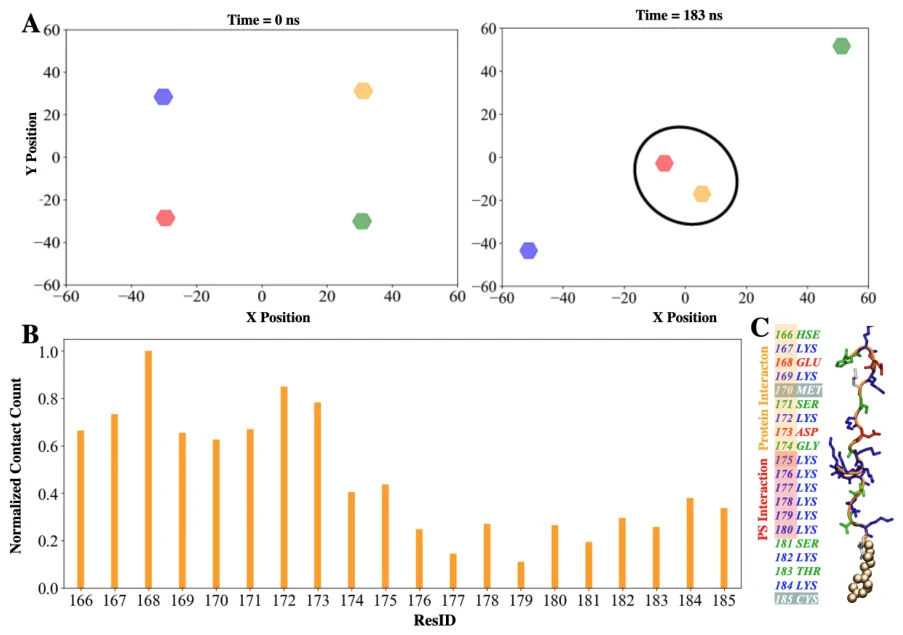
**HVR–HVR interactions in the membrane.** (**A**) In the initial simulation setup, the four HVRs (hexagons) are placed equidistant above the membrane, as shown in the 2D xy map of the membrane (**left**). During the simulation, the HVRs form dimers, though some of them persist only transiently (**right**). (**B**) The key dimeric interactions involve residues 166 to 175 according to the contact analysis between HVRs from all simulations. The contact count is normalized to the maximum number of counts. (**C**) Structural model of the HVR colored by residue properties (blue: basic, red: acidic, green: polar, white: hydrophobic) and indicated interaction sites for dimerization (protein interaction) and PS interaction.

Previous studies suggest dimerization or nanoclustering of K-Ras as a necessary step for signal transduction [71,72,73,74]. K-Ras-Raf dimerization is also believed to be necessary for signaling; therefore, K-Ras oligomerization is an active field of study. There are standing questions about the mechanistic details of dimer formation, the sequence of events, and protein association as the complex forms. Whether the dimerization is driven by K-Ras or its downstream effector, Raf, is unclear. In this study, we posit that the HVR–HVR interactions, most likely aided by anionic lipids, are a precursor to the whole protein dimerization. Throughout the trajectories, the HVRs form transient dimers (Figure 9A), exploring close distances to each other. The residues most involved in this interaction are located toward the N-terminus of the HVR (Figure 9B), with E168 forming the most contacts with other HVRs. This negatively charged residue may help drive the transient dimerization by interaction with the lysines not belonging to the poly-lysine chain. These interaction regions and their relationship to the PS-interacting region of the HVR are summarized schematically in Figure 9C. This exploration of a dimer state by the HVRs may lead to the dimerization process by bringing two K-Ras proteins close, followed by a stronger, more stable association of the G-domains.

### 3.3. HVR Restrains the G-Domain Orientations

**Figure 10:**
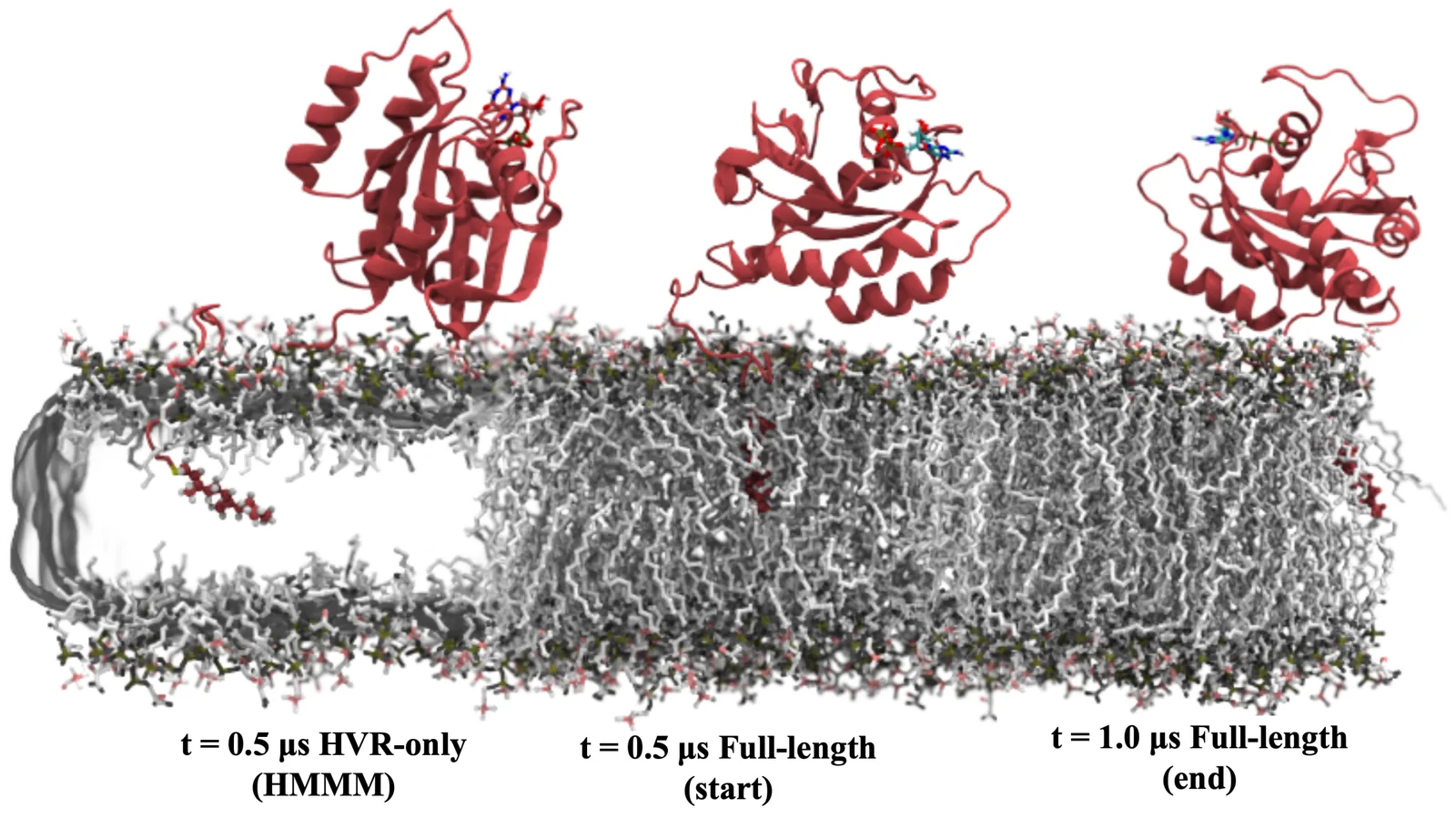
**Setup for full-length K-Ras simulations.** Shown are three frames from the simulation sequence. The initial HVR structure is obtained from the final frame of the HVR-only HMMM simulations at t=0.5μs, which was used to connect the G-domain (**left**) and initialize the full-length simulation system in a full-tailed membrane (**middle**). The (**right**) panel shows the final configuration after 0.5 μs of full-length simulation, at t=1.0μs.

As detailed earlier, the G-domain alone does not bind to the membrane in a consistent manner to interact with downstream effectors, but in the presence of the HVR, its binding seems to be largely affected by the way the HVR is anchored in the membrane (Figure 10). The HVRs show strong preferential binding to anionic lipids, suggesting they are the ones dictating the K-Ras-membrane contacts. Interestingly, the HVR is the portion of the protein that shows variation between different isoforms, while the G-domain is almost exactly the same between them.

When the G-domain is restricted by the HVR, the simulations only show a specific mode of membrane binding, with the residues of the allosteric lobe of the G-domain facing the membrane, leaving the orientation open for Raf binding.

**Figure 11:**
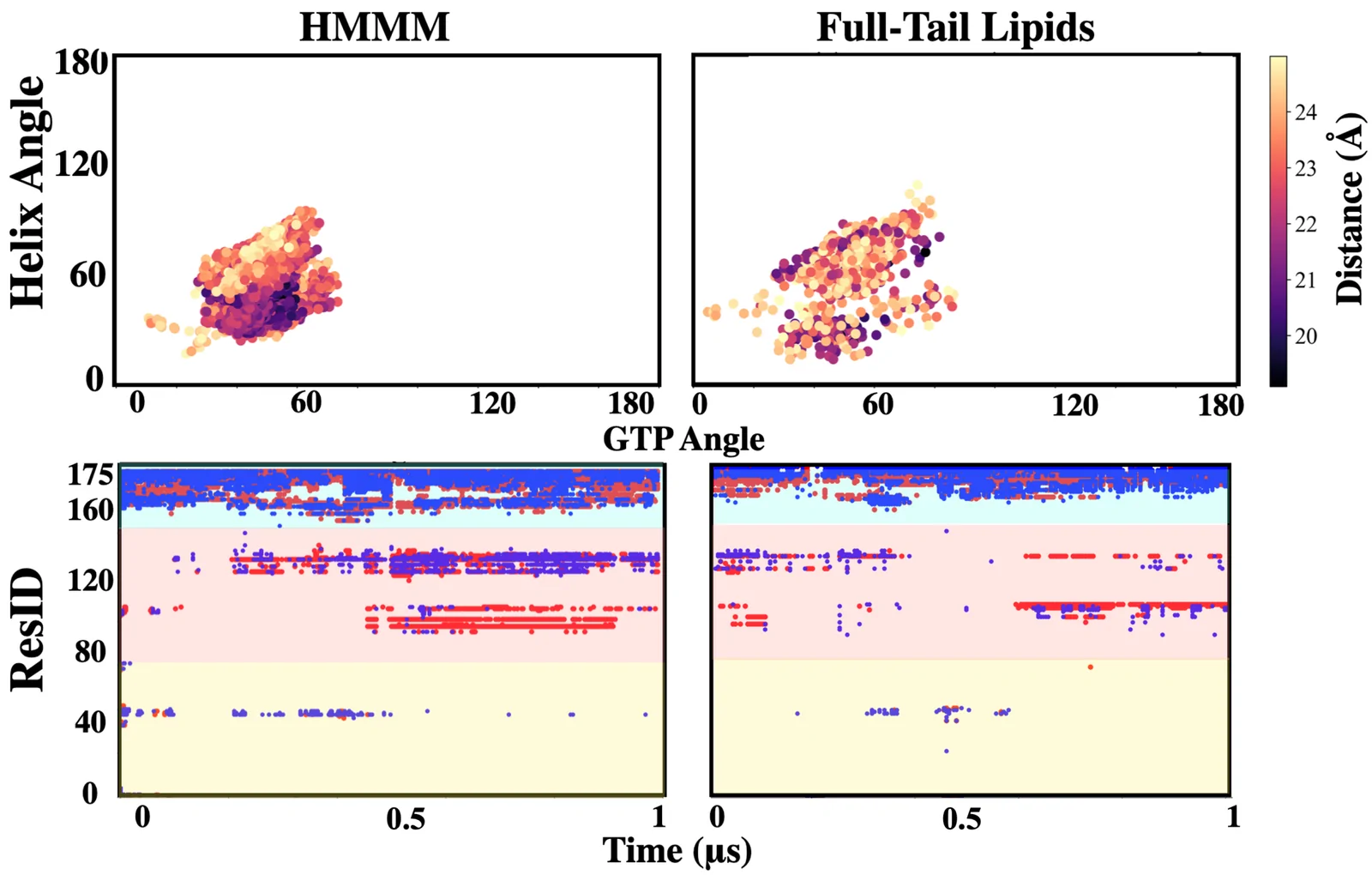
**Protein–lipid contacts and orientation behavior of full-length K-Ras.** (**Top**) panels: Orientation distributions of full-length K-Ras in HMMM (**left**) and full-tailed (**right**) membranes, shown in the space formed by the GTP and Helix angles. Each point is colored by the distance between the G-domain and the membrane surface (color bar, right). (**Bottom**) panels: Time progression of protein–lipid contacts for the same systems. Contacts with PC and PS lipids are indicated in blue and red, respectively. Residue positions are shown on the y-axis, with the effector lobe (yellow), allosteric lobe (pink), and HVR (cyan) highlighted. Lipid interactions occur predominantly in the HVR, with additional contacts observed through the allosteric lobe of the G-domain.

The G-domain undergoes binding and unbinding events, although persistently with residues in the allosteric lobe, and very few in the effector lobe. The HVR (residues 166–185) stays in the membrane, showing unperturbed contacts with lipids (Figure 11). The full-length K-Ras in the HMMM and full-tail membranes show broadly consistent orientational behavior, with the HVR remaining membrane-associated and the G-domain preferentially sampling orientations that expose the effector-binding region (Figure 11). Minor differences in the sampled orientations are nevertheless observed between the two membrane representations. The enhanced lipid mobility of the HMMM model facilitates membrane reorganization and accelerates sampling of protein–lipid interactions [75], whereas the full-tail bilayer provides more realistic lipid packing and hydrophobic-core properties. Consequently, the HMMM model is useful for efficiently sampling membrane-binding configurations, but its shortened lipid tails and artificial hydrophobic core do not fully reproduce the packing, viscosity, and dynamics of a physiological full-tail lipid bilayer. HMMM-derived membrane-binding and orientation preferences should therefore be interpreted primarily in terms of the sampled binding modes rather than as quantitatively equivalent to the dynamics of a physiological membrane. Importantly, validation of HMMM-derived membrane-binding configurations using full-tail membrane simulations is highly advised [75]. The overall agreement between the HMMM and full-tail systems in our simulations supports the robustness of the observed orientations, while the minor differences are consistent with the different lipid mobility and packing characteristics of the two membrane models.

**Figure 12:**
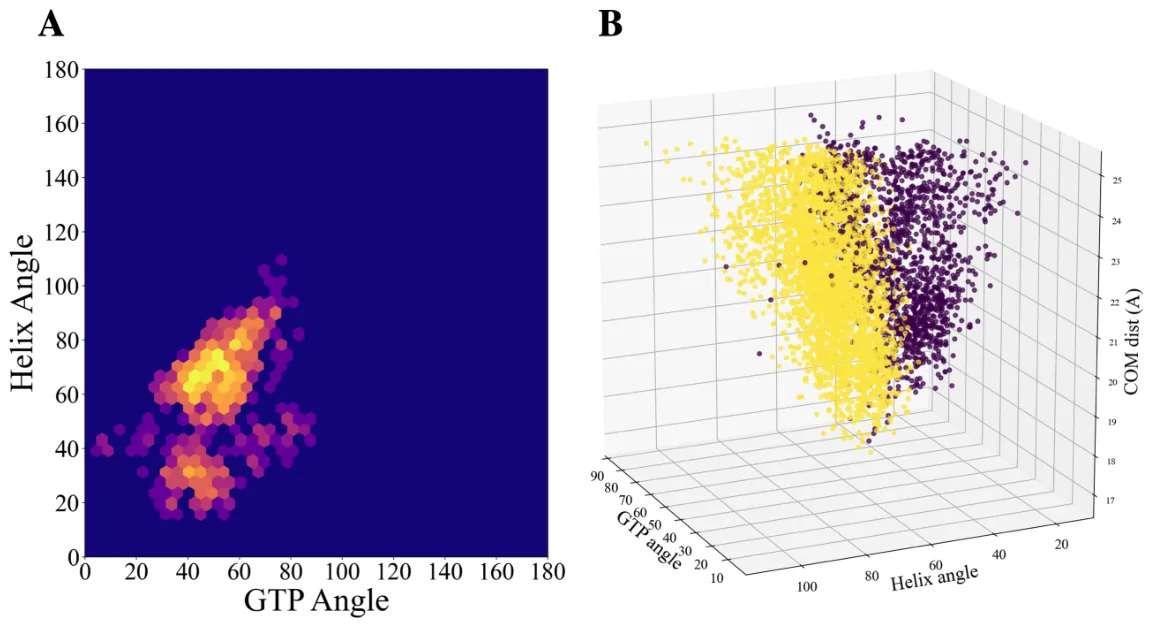
**Protein–membrane contacts of full K-Ras.** (**A**) Orientation histogram for full-length K-Ras, generated by concatenating angular data across all replicas. The color gradient from purple (low frequency) to yellow (high frequency) reflects the relative density of sampled orientations in the angular space defined by the Helix and GTP angles. (**B**) GMM clustering reveals two dominant membrane-bound orientations for full-length K-Ras.

**Figure 13:**
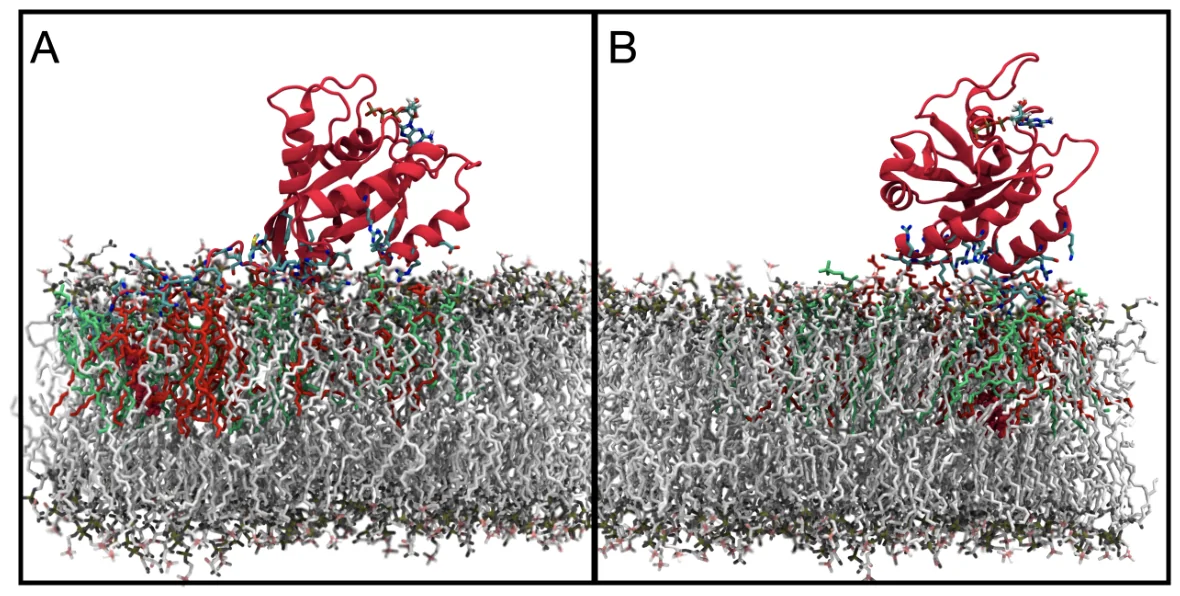
**Example bound states of full K-Ras.** One frame was chosen to represent each of the two clusters shown in Figure 12, based on the highest populated bins in the histogram for each cluster. The G-domain faces the membrane, making contact with the membrane either through the β1−β3 strands (**A**) or through the α3−α4 helices (**B**). The residues within 5 Å of the membrane are displayed explicitly using Licorice. The lipids within 5 Å of the protein are also colored (red for POPS and green for POPC).

A recent study detailing the interface between Ras and Raf1 Ras-binding domain cysteine-rich domain (RBDCRD) reports the key residues in Ras are located in the switch I region, specifically I24-R41 [76]. The orientations observed in this study for full K-Ras show these residues would be exposed to the solvent, allowing for Raf1 binding (Figure 12 and Figure 13). Previous studies with significantly more sampling found the G-domain to be restricted to two [23,24] or three [26,53] orientations on the surface of the membrane, although a third orientation was found to act as a transitional one between two main states or one in which the G-domain is not membrane bound [29]. Consistent with these observations, recent quantitative paramagnetic NMR analysis showed that KRAS4B lacking the HVR samples a broader range of membrane orientations, supporting a role for the HVR in restricting G-domain orientation [27]. Our full-length system echoes these results, showing the sampled orientation distribution (Figure 12A) and two dominant membrane-bound orientation clusters (Figure 12B). Together with the heterogeneous orientations observed for the isolated G-domain, this comparison supports a model in which HVR tethering restricts the orientational space accessible to the G-domain while maintaining solvent exposure of the effector-binding region.

## 4. Conclusions

K-Ras is one of the most frequently mutated Ras isoforms and accounts for a large fraction of oncogenic Ras mutations. While its G-domain is highly conserved relative to the other Ras isoforms, N-Ras and H-Ras, K-Ras differs substantially in its HVR, which contributes to membrane anchoring. The HVR therefore represents an important element in K-Ras membrane organization, although its highly dynamic and disordered nature makes it challenging to characterize structurally. The present systematic computational study of the different structural components of K-Ras provides insight into the contribution of the HVR to membrane binding and G-domain orientation.

Using an ensemble of conformations, we captured a broad range of orientations and distinct modes by which the isolated G-domain interacts with the membrane. Clustering identified seven major orientations, and contact analysis indicated that several of these orientations can reduce the accessibility of regions involved in downstream effector binding. The HVR, in contrast, exhibits preferential interactions with anionic lipids through its poly-lysine region. The hydrophobic M170 residue near the N-terminus of the HVR also acts as a secondary membrane anchor in a subset of the simulations. In the full-length K-Ras simulations, the G-domain samples a more restricted range of orientations, supporting a role for the HVR in constraining G-domain orientation at the membrane surface and thereby modulating the accessibility of the effector-binding region. In addition to its interactions with the membrane, the HVR forms transient associations with neighboring HVRs. Although these simulations do not establish a direct mechanism for K-Ras dimerization, such transient HVR–HVR contacts could increase the local proximity of membrane-bound K-Ras molecules and thereby facilitate subsequent protein–protein interactions.

## Data Availability

The simulation input files, structure files, parameters, and truncated MD trajectories have been deposited in a public repository [https://doi.org/10.5281/zenodo.15995392]. Simulation input scripts, analysis tools, and any custom code developed in this study are also available at this repository.

## Supplementary Materials

The following supporting information can be downloaded at: https://www.mdpi.com/article/10.3390/membranes16090304/s1, Table S1: Summary of the performed simulations; Figure S1: FVE for clustering; Figure S2: Orientation of the G-domain; Figure S3: Secondary structure of HVR 542.
