# Supplementary material for: Regulation of Membrane Association and Downstream Effector Interaction of K-Ras by Its Hypervariable Region
Source: Membranes (Basel). 2026 Sep 16;16(9):304. doi: 10.3390/membranes16090304 (PMC13609897; doi:10.3390/membranes16090304)
Supplement: Supplementary file 1 [file membranes-16-00304-s001.zip › membranes-4531102-supplementary.pdf]

Supporting Information

Regulation of Membrane Association and Downstream Effector Interaction of K-Ras by Its Hypervariable Region

Anda Trifan, Hossein Omidi Ardali
Josh V. Vermaas, Till Rudack, and Emad Tajkhorshid *

Theoretical and Computational Biophysics Group, NIH Resource forMacromolecularModeling and Visualization, Beckman Institute for Advanced Science and Technology, Department of Biochemistry, Center for Biophysics and Quantitative Biology, University of Illinois Urbana-Champaign, Urbana, IL 61801, USA
* Correspondence: emad@illinois.edu

Contents

Table S1: Summary of the performed simulations
Figure S1: FVE for clustering
Figure S2: Orientation of the G-domain
Figure S3: Secondary structure of HVR

Table S1. Summary of the simulations performed in this study

**Simulation**	**Time**	**# Proteins**	**# Replicas**	**Aggregate Time**
G-domains	1 μs	2 G-domains	6	6 μs
Linkers	0.5 μs	8 Linkers	3	1.5 μs
Full-Length/HMMM	0.5 μs	1 Full-Length	1	0.5 μs
Full-length/Full Membrane	0.5 μs	1 Full-Length	1	0.5 μs

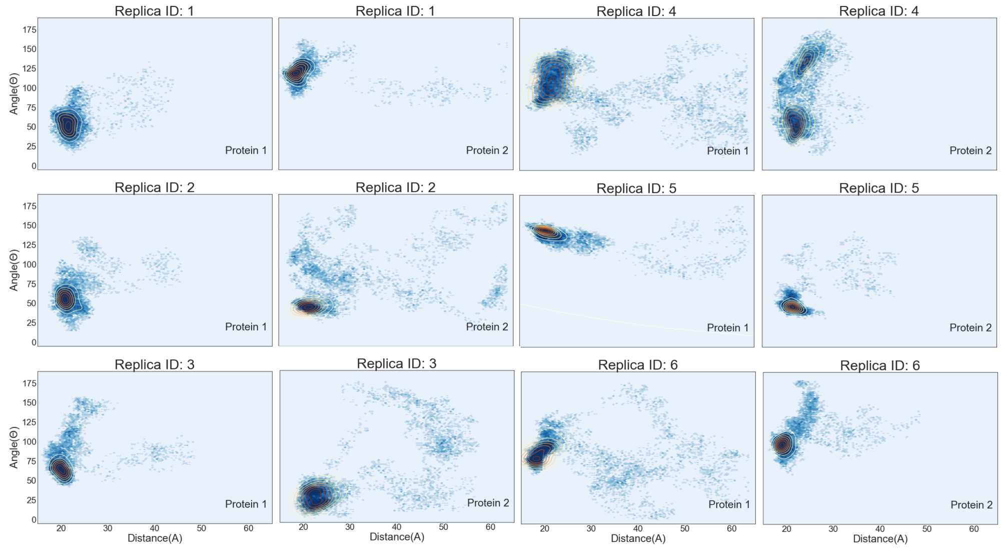

Figure S1. Orientation of the G-domain on the membrane as a function of distance from the membrane phosphate plane. The Angleθ is the angle between the normal of the phosphate plane and the vector between the center of mass of the G-domain and the center of mass of the GTP molecule. The contour lines highlight the increased frequency of that orientation. The angles with respect to the membrane vary between 10 and 180 degrees, sampling almost all angles in between. All replicas bound to the membrane, some diffusing in solution for longer (ex. Replica ID:4, Protein1), or oscillating between two binding modes (i.e, Replica ID:4, Protein 2).

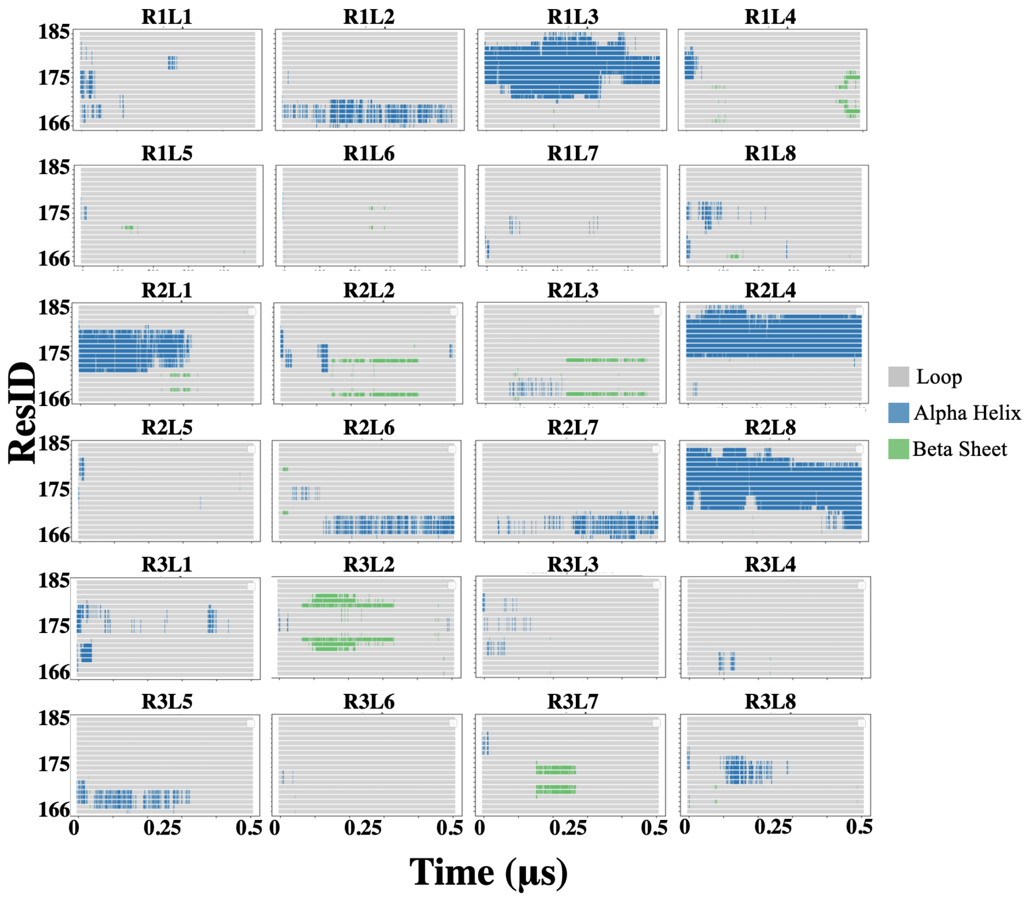

Figure S2. Secondary structure progression of the membrane bound HVR. The HVR structure itself is highly dynamic and did not exhibit a preferred structural motif stabilized by the membrane as revealed by the progression of the secondary structure (random coil: gray, *alpha*-helix: blue, *beta*-sheet: green) during the simulation. Most simulations show unstructured random coil structures, three simulations show stable helical content, however all three for different residue regions.

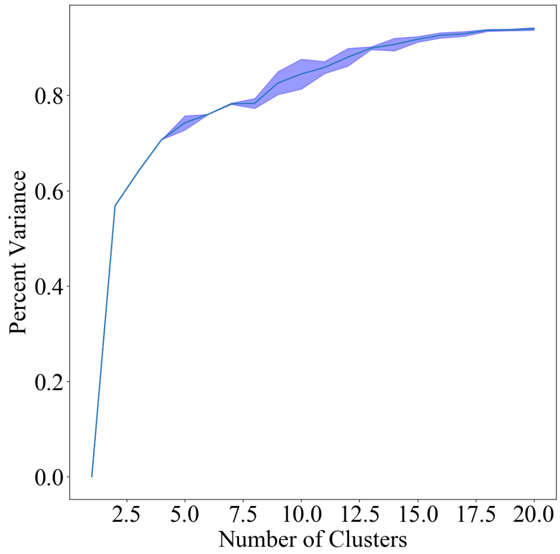

Figure S3. The FVE (fraction of variance explained) was calculated for up to k=20 clusters. Ten replicas were calculated to obtain an average and standard deviation of the knee curve. The kink in the curve, which in this case is observed 7, represents an appropriate number of clusters for the Gaussian Mixture Model clustering algorithm.
